# Aligned perylene bisimide hydrogels: a 3D printing approach to mechanophotoconductive films

**DOI:** 10.1039/d6ma01064d

**Published:** 2026-10-09

**Authors:** Rebecca E. Ginesi, Ziyao Xu, Tom Jacquin, Katarzyna Kozioł, Morteza Amjadi, Emily R. Draper

**Affiliations:** a School of Chemistry, University of Glasgow Glasgow G12 8QQ UK Rebecca.Ginesi@glasgow.ac.uk; b Centre for the Cellular Microenvironment, James Watt School of Engineering, Advanced Research Centre, University of Glasgow 11 Chapel Lane Glasgow G11 6EW UK; c James Watt School of Engineering, University of Glasgow G12 8QQ UK

## Abstract

Organic electronics often require molecular alignment to facilitate efficient charge transport. However, existing alignment methods (*e.g.*, magnetic alignment, dielectrophoresis, spin coating, and doctor blading) are energy-intensive, incompatible with water-based materials, or wasteful. Here, we demonstrate that extrusion-based 3D printing induces significant shear alignment in aqueous perylene bisimide (PBI) and poly(ethylene oxide) (PEO) blends, eliminating the need for external fields or incompatible coating techniques. Upon drying, the resultant xerogels retain this alignment, yielding homogeneous, conductive, flexible films. These films exhibit robust mechanophotoresponsive behaviour: bending the aligned fibres to a radius of 45 mm enhanced the photocurrent response by 2.5-fold compared to the unbent state. Furthermore, the films maintained structural integrity over 1000 bending cycles, retaining 50% of their photoresponse. This work establishes 3D printing as an accessible route to fabricate aligned, water-processed organic materials for wearable mechanophotoresponsive applications.

## Abstract 2

Water-based 3D printing uses extrusion shear to align self-assembled perylene bisimide/polymer hydrogels, producing homogeneous conductive xerogel films that exhibit mechanophotoresponsive behaviour and retain conductivity after 1000 bending cycles.

## Graphical abstract

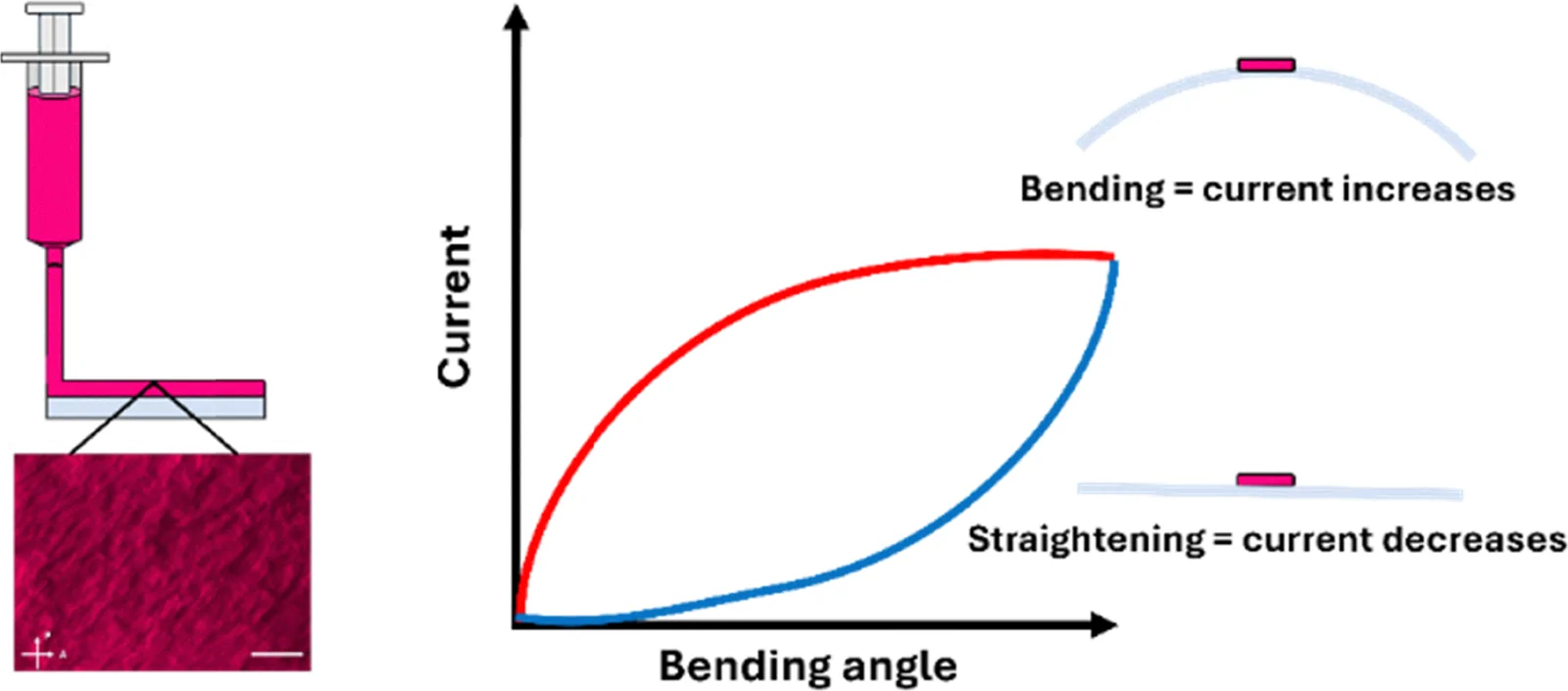

## Introduction

There is considerable interest in flexible conductive materials for the development of “smart materials”, driven by their potential applications in wearable electronics.^1,2^ Flexibility can describe the bendability, resilience, or stretchability of a material.^3^ Such properties are highly desirable for applications such as robotic skin and wearable biological sensors.^4,5^ In particular, robotic skin requires materials capable of sensing parameters including pH, moisture, and temperature, which remain challenging to achieve using conventional metal- or silicon-based electronics.^6^ Additionally, many crystalline electronic materials are intrinsically brittle, limiting their suitability for flexible devices. By contrast, self-assembled small molecules, while often amorphous, can form monodisperse and flexible fibrous structures that are well-suited to mechanically compliant electronics. For example, Pandit *et al.* developed a flexible organic photodetector based on self-assembled, crystalline thin films of 6,13-bis(triisopropylsilyethynyl) pentacene (TIPS-Pentacene).^7^ The device could withstand thousands of repeated bending/folding cycles and regain its initial photoresponse characteristics if left to rest for 2 hours. Self-assembled dipeptides have also been studied, as seen in the work of Ravikumar *et al.*, who utilised self-assembled diphenylalanine (FF) analogues in piezoelectric nanogenerators.^8^ The self-assembled FF nanostructures could be tuned by the addition of chemical linkers to enhance their piezoelectric outputs. By tuning the self-assembled nanostructures, this method opens a range of applications for powering implantable devices. Other small organic molecules (such as amino acids) have been used in biomedical applications, including chip sensors and ultrasound transducers.^9,10^

Amino acid-appended perylene bisimides (PBIs) have emerged as promising candidates due to their favourable optoelectronic and mechanical properties.^11–14^ These systems are thermally robust, air-stable, moisture-insensitive, readily processable, and exhibit a remarkably long-lived radical species.^15–17^ Furthermore, they can be used to form mechanophotoresponsive films,^6^ in which the electrical response varies systematically with applied strain.

For practical implementation in organic electronics, control over structural anisotropy is crucial, as fibre orientation strongly influences charge transport and device performance. In addition to alignment, materials must exhibit suitable exciton diffusion lengths and a controlled spatial arrangement of self-assembled fibres over device-relevant length scales.^18^ Many methods of alignment exist, such as spin-coating,^19,20^ magnetic-field-assisted alignment,^21^ dielectrophoresis,^22^ and doctor blading.^23^ While magnetic fields can generate highly aligned, directionally dependent films, their use typically requires specialised and costly infrastructure. Moreover, reproducibility can be sensitive to environmental conditions such as temperature and humidity, particularly when solvent evaporation governs structure formation.

Low-cost approaches, such as spin-coating and doctor blading, offer greater accessibility but can be wasteful and difficult to control.^21^ Furthermore, using such techniques on low-viscosity, water-based solutions is typically unsuccessful, with slow solvent evaporation hindering effective fibre alignment. Consequently, there is a need for scalable and controllable methods that enable alignment in aqueous, self-assembled materials without reliance on large magnetic fields or wasteful coating techniques.

Here, we address this challenge using PBI-A (Fig. 1a), a well-studied low-molecular-weight gelator (LMWG) within our group.^11,14,24^ Its perylene core promotes strong π–π interactions, leading to well-defined nanostructures, while the alanine substituents influence molecular packing, allowing control over morphology and electronic properties. Previously, we have demonstrated that the addition of a non-gelling polymer additive creates hydrogels suitable for 3D printing applications.^25^ These printed hydrogels were extensively studied using rheology and small-angle neutron scattering (SANS) to better understand the impact of printing on the bulk properties of the hydrogels. Here, we expand on this work to demonstrate the formation of aligned and flexible materials *via* 3D printing of amino acid PBI/polymer-based hydrogels (Fig. 1b). Upon drying, the resulting xerogels retain this alignment due to the shear applied during printing, producing flexible films with improved homogeneity. These aligned materials are also mechanophotoresponsive, showing an increase in photoresponse upon bending strain. This approach establishes 3D printing as a versatile and accessible strategy for aligning water-based, self-assembled electronic materials for potential application in wearable sensors and flexible electronic devices.

**Fig. 1:**
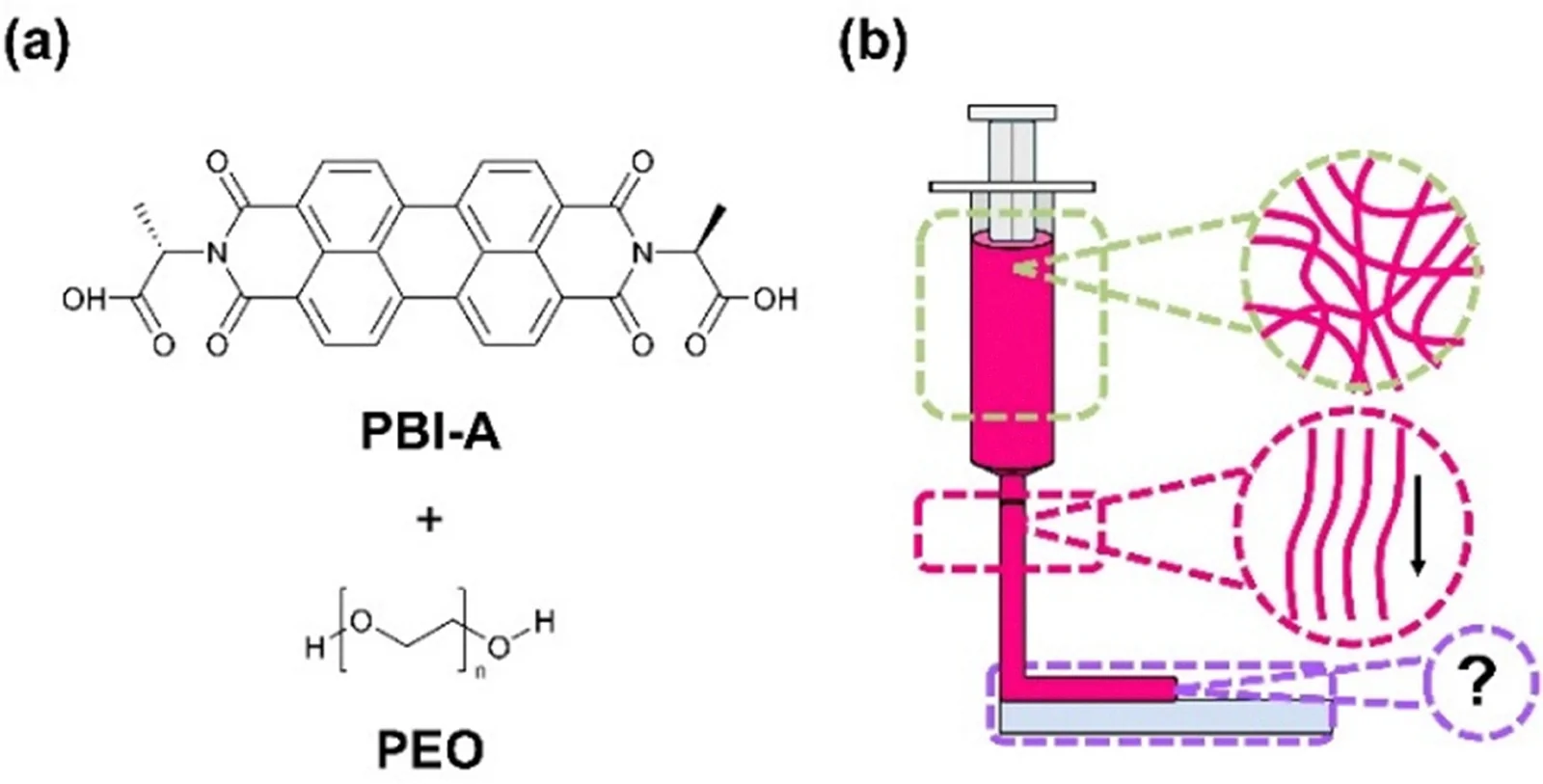
(a) Structures of PBI-A and PEO used in this study. (b) Cartoon demonstrating how extrusion-based 3D printing could be used as a method to create alignment in hydrogels and their resulting xerogels.

## Results and discussion

In our earlier work, we demonstrated that the addition of the non-gelling polymer poly(ethylene oxide) (PEO) to PBI-A creates a well-characterised hydrogel with tunable rheological properties suitable for extrusion-based 3D printing, while preserving the self-assembled PBI network.^25^ More recently, we compared PBI-A/PEO with blends containing several other polymers (poly(vinyl alcohol)), hyaluronic acid, and poly(oxyethylene) (40) stearate and found that, under identical printing conditions, none exhibited the same combination of printability and alignment (Fig. S1–S3). These observations further support the use of PBI-A/PEO as a model system for investigating printing-induced alignment. Here, we therefore employ this established PBI-A/PEO platform to investigate the role of printing-induced shear in controlling fibre alignment and the functional properties of the resulting xerogel materials. For simplicity, the gel will be referred to as Gel-1 or Printed Gel-1, for non-printed/drop-casted and printed hydrogels/xerogels, respectively.

Cyclic voltammetry was first used to determine if the printing process impacted the reduction potential of the gel in a two-electrode window cell setup (Fig. S4 and Table S1). Gels were either prepared in or printed directly onto the fluorine-doped Sn oxide (FTO) glass. The reduction potentials were −0.12 V and −0.10 V for non-printed and printed gels, respectively. The shapes of the CV voltammograms for non-printed and printed gels were significantly different. There are two possible explanations for this change. The shape of the CV waveform changes depending on how electrons move through the system. Furthermore, as structures become larger, they should become less efficient at electron transfer due to a change in the available surface area. Therefore, the differences in CV waveforms could be due to variations in the micellar structures and/or nanostructures caused upon extrusion. The second explanation could be related to how the samples were prepared in the FTO window setup. For the non-printed gels, gelation occurred *in situ* directly on the FTO glass. In comparison, the printed gels were prepared in a syringe and printed onto the FTO glass. Therefore, there may be poorer contact between the glass and the printed gel, which could explain the less defined peaks and curve.

We have previously shown that amino acid-appended PBIs can form photoconductive xerogels.^18,26^ Printed gels were dried to form xerogels and imaged using cross-polarised optical microscopy to investigate how printing impacted the xerogel morphology (Fig. 2). These gels were compared to xerogels formed by drop casting in a 0.5 × 6 cm mould (Fig. 2a). The printed gels showed alignment (Fig. 2b), which is promising if these materials are to be used in optoelectronic devices. Aligned fibres facilitate electron transport, as the path is shorter, reducing the chance of charge recombination.^27^ These results suggest that the alignment caused by the shear applied during printing remains after drying, which can sometimes be lost when using doctor blading or spin-coating of water-based materials. In comparison, the xerogels formed by drop-casting showed no alignment. Scanning electron microscopy (SEM) analysis further confirmed these observations, again showing alignment of the fibres in the printed xerogels (Fig. 2b and Fig. S7). Drop-casted xerogels displayed a predominantly isotropic morphology (Fig. 2a). Together, the optical microscopy and SEM data demonstrate that extrusion-induced shear generates alignment within the PBI network that is maintained after drying and is observable across multiple length scales.

**Fig. 2:**
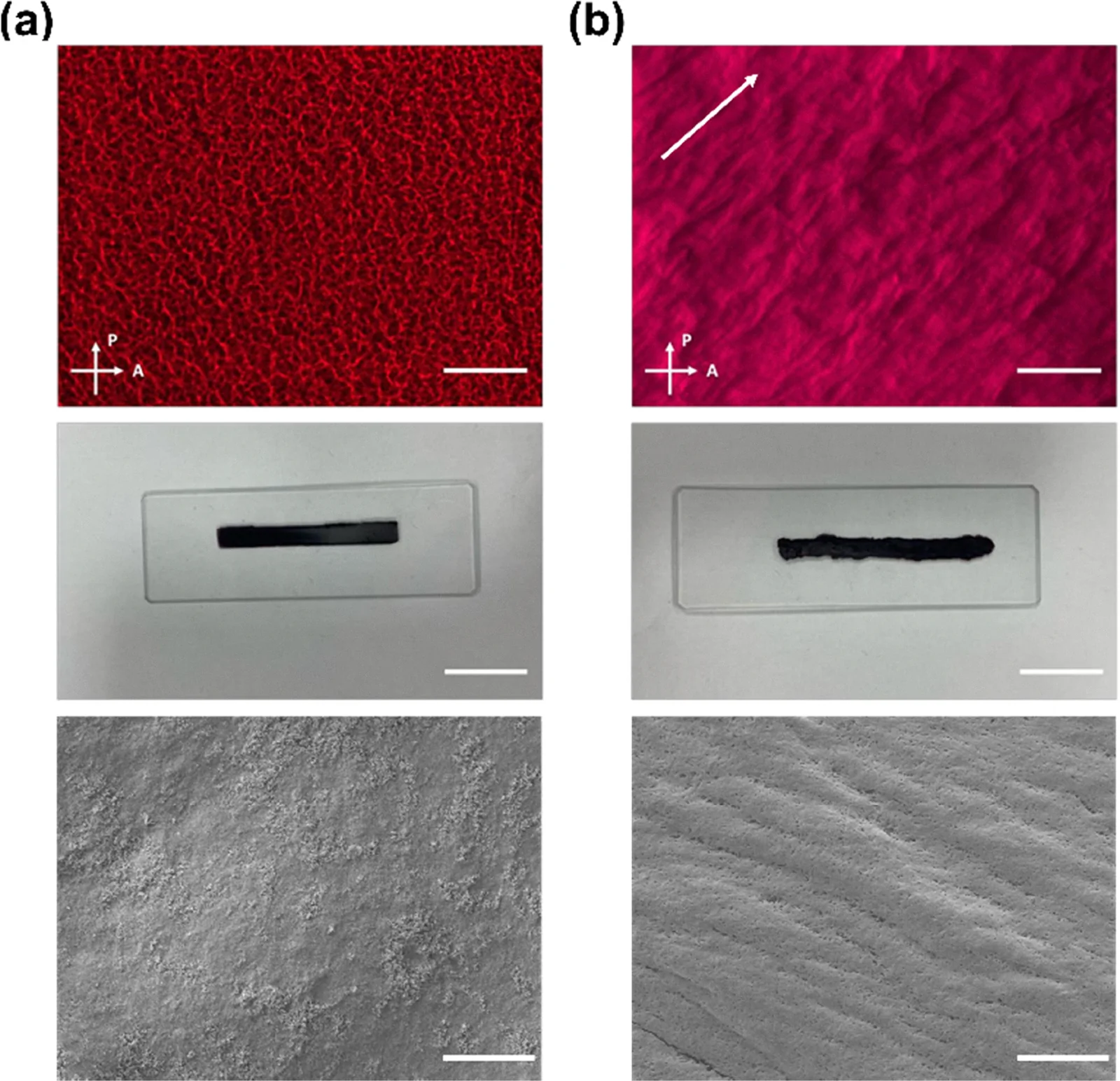
Cross-polarised optical microscopy images (top), photographs (middle), and SEM images (bottom) of (a) Gel-1 and (b) Printed Gel-1 xerogels. SEM images were acquired at 5.00k× magnification. Scale bar represents 100 µm for the optical microscopy images, 2 cm for the photographs, and 10 µm for the SEM images. In (b), the arrow indicates the alignment direction.

For the printed gels to be suitable for applications (such as optoelectronic devices), the gels should be homogeneous along the printing axis. Therefore, cavitation rheology was utilised to measure the critical pressure at different points along the printed gel (Fig. S8). This technique is a form of microrheology which utilises the cavitation effect to probe the localised mechanical properties of a material.^28^ Cavitation rheology has the advantage that it can be conducted on gels of any shape in their native environment.^29,30^ Briefly, the method involves growing a cavity at the tip of a needle and quantifying a critical pressure of mechanical instability.^31^ The pressure at which this instability occurs is directly related to the modulus of the material, meaning cavitation rheology can easily probe the homogeneities of the gel. The critical pressure was measured in 0.5 cm increments along the length of Printed Gel-1, with a control experiment performed in a Sterilin vial, Gel-1 (Fig. S8). For Printed-Gel 1, the critical pressure was identical at points 1.0 and 1.5 cm along the printing axis (22 Pa for 1.0 and 1.5 cm, respectively) and was slightly lower (16 Pa) at 0.5 cm along the gel. This decrease in pressure could be due to this section of gel being from the top of the syringe, so it is not as compressed as the gel from the middle and bottom of the syringe. The critical pressure of Printed Gel-1 was slightly lower than that of Gel-1 (20 Pa *versus* 23 Pa for Printed Gel-1 and Gel-1, respectively), suggesting printing changes the gel's mechanical properties.

Profilometry was performed on the resulting Printed Gel-1 xerogels to ensure this homogeneity was preserved upon drying (Table S2 and Fig. S9). It is important that the sample thickness is consistent across the gel to ensure the photoresponse is the same. Furthermore, if the films are too thick, this could prevent light penetration through the gel. The thickness was measured by taking profilometry measurements at 0.5, 1.0, and 1.5 cm along the printing direction, with very subtle differences in thickness (thicknesses ranging between 16.60 and 16.85 µm). Such results agree with the cavitation rheology data (Fig. S8). Furthermore, nanoindentation measurements (Fig. 3 and Fig. S10) of Printed Gel-1 showed that the indentation modulus (EIT) values were similar along the printing axis (Fig. 3a). There were some variations in the indentation hardness (HIT) values at the top and bottom of the xerogels (Fig. 3b), which could be due to slight compression inside the syringe. Nanoindentation was also attempted on Gel-1 xerogels to determine if printing impacted the elasticity or hardness. However, no measurements could be taken due to the inhomogeneity of these xerogels and the presence of cracks (Fig. S11).

**Fig. 3:**
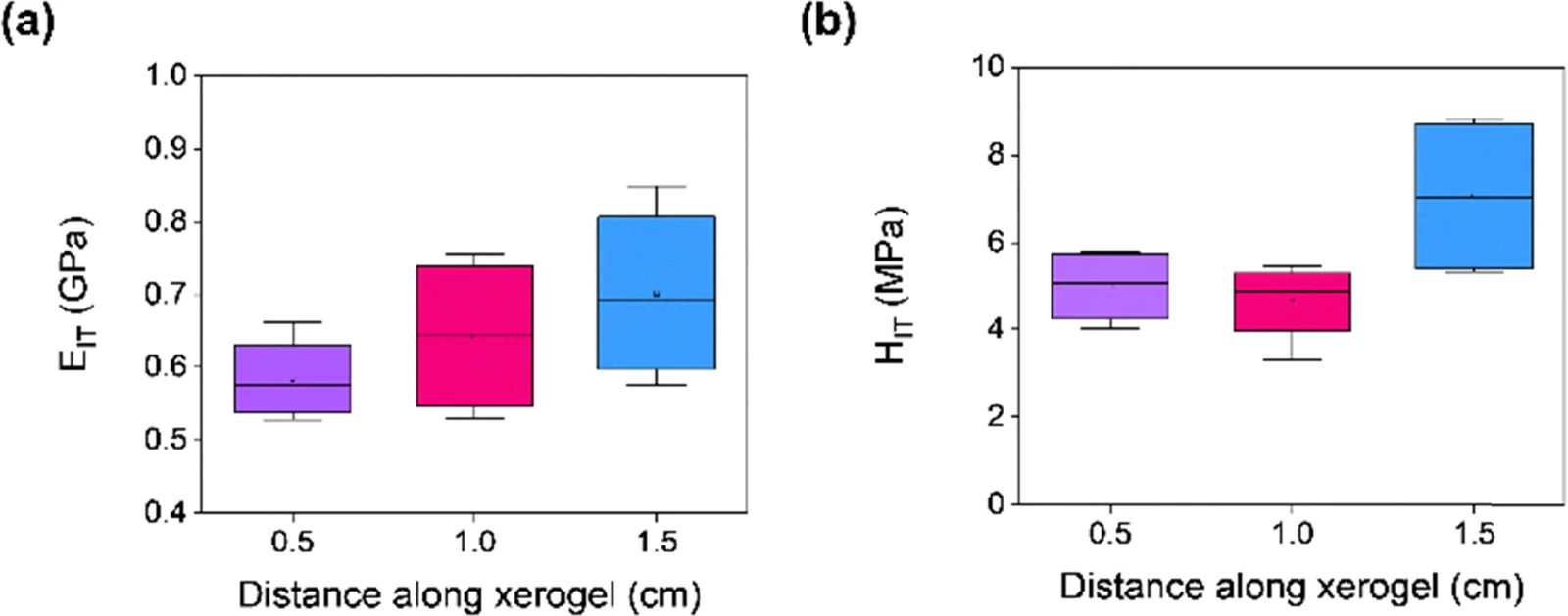
The average (a) *E*_IT_ and (b) *H*_IT_ of Printed Gel-1 xerogels along the printing axis. Measurements were run in triplicate, with error bars representing standard deviation.

To quantify the electrical properties of the printed xerogels, the DC conductivity of the printed films was measured using a four-point probe (Table 1). The printed xerogels exhibited a conductivity of 970 ± 10 µS m^−1^. The presence of electrical conductivity after drying demonstrates that the interconnected PBI network is retained within the xerogel structure.

**Table 1:** DC electrical properties of Printed Gel-1 xerogels measured using a four-point probe method. The sheet resistance, resistivity, and conductivity of the dried printed films are shown. Values are reported as mean ± standard deviation (*n* = 3)

	Sheet resistance (MΩ square^−1^)	Resistivity (kΩ m)	Conductivity (µS m^−1^)
Printed Gel-1	81.9 ± 1.5	1.4 ± 0.3	971.6 ± 9.7

To test the flexibility of the printed xerogels, hydrogels were printed onto 120 micron thick acetate sheets to allow for bending of the substrate. These were compared with xerogels drop-cast from PBI-A only solutions to determine if both printing and the polymer are required for flexibility. Previous work on PBI-A has shown that there is no difference in the adsorption of the material on glass compared to plastic.^6^ Chronoamperometry measurements were collected under various bending angles (Fig. 4). The length of plastic used for each xerogel was the same to ensure each film had the same radius of curvature upon bending (Fig. 4a). The measurement of the effect of the degree of curvature on the resistance of the films was conducted under constant irradiation with a 365 nm LED. From previous EPR and UV-vis absorption measurements on amino acid-appended PBIs, it was assumed that the sample was saturated with radical anion, meaning any changes in current would be due to bending.^6,16^ The films were bent to a maximum bending angle of 70° and straightened back to 0° whilst continuously measuring the current.

**Fig. 4:**
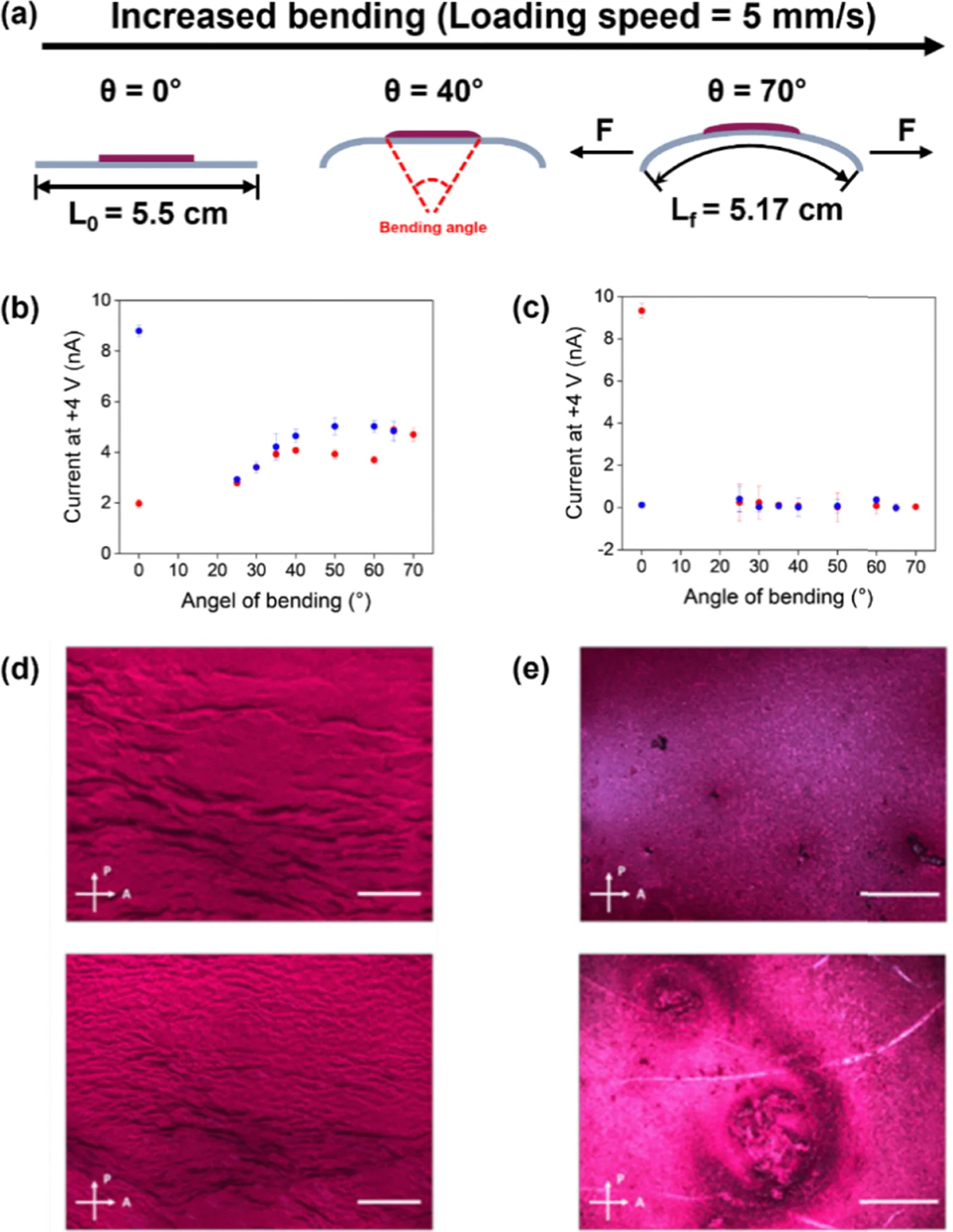
(a) Schematic illustration of the flexible device under increased bending load from 0° to 70° at a constant displacement speed of 5 mm s^−1^, with an initial chord length (*L*_0_) of 5.5 cm and a final chord length (*L*_f_) of 5.17 cm. *F* represents force and *θ* represents bending angle. (b) and (c) Current at +4 V whilst bending (blue) and straightening (red). Measurements were run in triplicate, with error bars representing standard deviation. (b) Printed Gel-1 and (c) PBI-A xerogels. (d) and (e) Cross-polarised optical microscope images of (d) Printed Gel-1 and (e) PBI-A xerogels before (top) and after (bottom) bending. Scale bars represent 100 µm.

The bending strain was systematically calculated using *ε* = ±*h*/*r*,^32^ where *h* is the distance from the structural neutral axis of the bilayer system to the centreline of the functional film (≈68.3 µm), and *r* is the corresponding radius of curvature (Table 2). The *r* value has a relationship with the chord length (*c*) as: *c* = 2*r* sin(*l*/2*r*), where *l* is the arc length of the sample (55 mm). Comprehensive derivation steps and mechanical assumptions are detailed in the supplementary information (SI) under the bending strain subheading.

**Table 2:** Summary of calculated geometric and mechanical bending parameters for the films across evaluated deformation angles. Values are derived using an initial sample arc length (*l*) of 55 mm and a calculated distance from the structural neutral axis to the functional layer centreline (*h*) of 68.3 µm

Bending angle [°]	Radius of curvature [mm]	Chord length [mm]	Bending strain [%]
0	∞	55.00	0
25	126.1	54.57	0.054
30	105.0	54.37	0.065
35	90.0	54.15	0.076
40	78.8	53.89	0.087
50	63.0	53.27	0.108
60	52.5	52.52	0.130
70	45.0	51.64	0.152

The Printed Gel-1 xerogels maintained electrical continuity during the measurements (Fig. 4b). Bending the films increased the current, whilst straightening the films decreased the current. However, once straight, the current produced was greater than the original value. Furthermore, the films did not show cracking under the microscope after bending (Fig. 4d). Xerogels of PBI-A alone showed a large decrease in current upon bending (Fig. 4c), which did not recover upon straightening. Inspection of these xerogels under the microscope showed that cracks formed after bending (Fig. 4e). Such results suggest that the polymer and alignment caused by printing could aid in the xerogels’ response to bending. The aligned fibres of the Printed Gel-1 xerogels could act like layers that could slide over each other, stretching the film upon bending and creating longer pathways for the current and thus increasing the resistivity.^6,33^ Furthermore, energy dissipation could more easily move across fibres when bent. Moreover, the more uniform surface of the Printed Gel-1 xerogels could be less prone to cracking with bending.^6,34^ Similar results have been observed with macroscopically aligned fibrous PBIs, which showed improved mechanical properties and flexibility compared to isotropic or non-fibrous films.^35^

Printed Gel-1 xerogels underwent 1000 bending cycles to test their electromechanical stability under repeated mechanical strain (Fig. S12 and S13). This cycle count aligns with common literature standards (500–1500 cycles) for assessing the fatigue resistance of flexible conductive materials.^36–39^ After 1000 cycles, the films continued to exhibit electrical conductivity (Fig. S13), successfully sustaining a current despite the repeated deformation (0.05 µA and 0.03 µA before and after bending cycles, respectively). The observed deviation from linearity after repeated bending is likely associated with the formation of microscopic defects or cracks within the xerogel film, which alter the current pathways. Importantly, the films retain a continuous current output, demonstrating high tolerance to cyclic bending. This suggests that while the conductive network is modified through bending, the overall electrical pathways remain unbroken.

## Conclusions

In conclusion, we have demonstrated that extrusion-based 3D printing serves as a highly effective and accessible strategy for inducing alignment in aqueous PBI-A/PEO hydrogels. Unlike energy-intensive magnetic or mechanical alignment techniques, this approach exploits printing-induced shear to organise self-assembled fibres, a structural anisotropy that is successfully preserved in the resulting xerogel films.

The combination of the PEO polymer matrix and the shear-aligned PBI-A network yields films with improved mechanical and electromechanical properties compared to drop-cast films. Specifically, the printed films exhibited flexibility and reversible mechanophotoresponsiveness, with the current produced being a function of bending angle. To evaluate long-term durability, we subjected the films to a 1000-cycle bending test. The films maintained conductive integrity throughout, successfully sustaining a current after the final cycle. These results establish 3D printing as a viable, low-waste manufacturing route for water-processed, wearable flexible organic electronic devices.

## Author contributions

R. G. and E. D. conceptualised the study; R. G., Z. X., T. J., K. K., M. A., and E. D. developed the methodology; R. G. validated the results and performed the formal analysis; R. G., Z. X., T. J., K. K., M. A., and E. D. conducted the investigation; R. G. managed data curation and visualisation; M. A. and E. D. supervised the project; E. D. oversaw project administration and secured funding. All authors contributed to writing the manuscript.

## Conflicts of interest

There are no conflicts to declare.

## Supplementary Material

MA-007-D6MA01064D-s001

## Data Availability

The data supporting this article have been included as part of the supplementary information (SI). Supplementary information is available. See DOI: https://doi.org/10.1039/d6ma01064d.
