# Supplementary material for: Aligned perylene bisimide hydrogels: a 3D printing approach to mechanophotoconductive films
Source: Mater Adv. 2026 Oct 9. Online ahead of print. doi: 10.1039/d6ma01064d (PMC13650769; doi:10.1039/d6ma01064d)
Supplement: Supplementary file 1 [file MA-OLF-D6MA01064D-s001.pdf]

Supplementary Information (SI) for Materials Advances.
This journal is © The Royal Society of Chemistry

Aligned perylene bisimide hydrogels: A 3D printing approach to mechanoresponsive films

Supporting Information

Rebecca E. Ginesi,[a,b*] *Ziyao Xu,*[a] *Tom Jacquin,*[c] *Katarzyna Kozioł,*[c] *Morteza Amjadi,*[c] *and Emily R. Draper*[a*]

[a]School of Chemistry, University of Glasgow, Glasgow, G12 8QQ, UK.

[b]Centre for the Cellular Microenvironment, James Watt School of Engineering, Advanced Research Centre, University of Glasgow, 11 Chapel Lane, Glasgow, G11 6EW, UK.

[c]James Watt School of Engineering, University of Glasgow, G12 8QQ, UK.

[*] Rebecca.Ginesi@glasgow.ac.uk; Emily.Draper@glasgow.ac.uk

Contents

Experimental Methods S2

Supplementary Figures and Tables S9

References S16

Experimental Methods

Materials. **PBI-A** was synthesised as previously reported.[1] All commercial reagents were used as received. Perylene-3,4,9,10-tetracarboxylic dianhydride (PTCDA), L-alanine, imidiazole, and poly(ethylene oxide) (PEO, average M_n = 500,000) was purchased from Sigma-Aldrich.

Preparation of PBI-A/PEO blends. Stock solutions of **PBI-A** and PEO were made separately in deionised water at concentrations of 20 mg/mL and 6.66 mg/mL for **PBI-A** and PEO, respectively. For **PBI-A** solutions, an equimolar equivalent of sodium hydroxide (0.1 M, aqueous) was added. The solutions were agitated on a MX-T6-S tube roller (SCILOGEX) overnight until all the gelator had visibly dissolved. The **PBI-A** solution was then adjusted to pH 9 using NaOH (0.1 M, aqueous). 2.5 mL of **PBI-A** and 7.5 mL of PEO were combined to give a blend with final concentrations of 5 mg/mL for each component. The blends were left to mix overnight.

pH measurements. An FC200 pH probe from HANNA instruments with a 6 mm x 10 mm conical tip was used for pH measurements at 25°C. The state of accuracy of the pH measurements is ± 0.1.

Preparation of PBI-A/PEO hydrogels. A pH-switch method was used to form the hydrogels. Solutions and blends were prepared as above. For non-printed hydrogels, 2 mL of solution was transferred to a 7 mL Sterilin vial containing a pre-weighed amount of glucono-*δ*-lactone (GdL, 12.5 mg/mL) and shaken three times. The sample was left to stand overnight to allow gelation to occur. For printed hydrogels, the sample was prepared in Sterilin vials as above, and then quickly

transferred to 3.5 mL syringes. The syringe nozzle was tightly wrapped in parafilm to prevent the gel from drying out. Syringes were stood vertically on their plungers and left overnight to gel.

3D printing. Gels were prepared in 3.5 mL syringes as above. A 3D printer (RepRap Ormerod 1) was modified and repurposed for gel printing by Dr Bart Dietrich (University of Glasgow).[2] The inner diameter of the 3.5 mL syringe nozzles used for extrusion was 2.2 mm. To print a 6 cm gel line at a shear rate of 2500 s^{-1} and a rate of extrusion of 166 μL/cm, the speed of the printer head was 9408 mm/min, raised 3 mm above the printing bed.

Cavitation rheology. Cavitation experiments were performed using a custom-built cavitation rheometer (Dr Bart Dietrich, University of Glasgow). The setup involves a syringe pump (World Precision Instruments Aladdin, AL-1000), a 50 mL syringe (BD Plastipak™), a needle of inner diameter 0.41 mm (Fisher Scientific, Hamilton™ Kel-F Hub Blunt Point Needle with Leur-lock attachment), the custom-made Cavitation Rheology Analyser Box (CRAB), a conductivity probe circuit for accurate homing of the 3D printer platform z-axis, a modified 3D printer platform for needle positioning, and a computer with "PuTTY" software to control the 3D printer platform and record pressure data from the CRAB.[3, 4] All these components were connected using plastic tubing and plastic adapters to allow air to be pushed through the system and create pressure. CRAB pressure readings were calibrated using a commercial manometer before measurements were recorded. Hydrogels were printed on glass microscopic slides (Thermo Scientific). For printed gels, measurements were taken

0.5, 1.0, and 1.5 cm along the length of the printed gel lines. The needle was homed to the surface of the slide, raised above the sample, and then lowered into the sample to a height of 0.5 mm above the slide surface to ensure constant sample depth between the slide and needle. This eliminated any issues due to inhomogeneities in the gel surface. The syringe pump was then turned on, with a pump rate of 0.4 mL/min. The experiment was finished after a drop in pressure was recorded. All measurements were carried out in triplicate.

Xerogel formation. For printed xerogels, a 6 cm line was printed as described above onto a glass microscope slide (Thermo Scientific) and left to dry in air overnight (average lab temperature 23.4°C and average lab humidity 31%). For non-printed xerogels, 2 mL of the **PBI-A**/PEO blend was transferred to a 7 mL Sterilin vial containing a pre-weighed amount of GdL (12.5 mg/mL) and gently shaken three times. 10 μL of the solution was drop cast inside a 3 x 3 mm mask and left to air dry overnight.

Optical microscopy. Cross-polarised light optical images were collected using a Nikon Eclipse LV100 microscope with a Nikon Plan ELWD 50x/0.60 or x10 lens attached to an Infinity2-1C camera. No post-processing was applied to the images.

Scanning electron microscopy (SEM). Prior to imaging, the samples were coated with 10 nm layer of 80% gold/20% palladium alloy to dissipate the sample charging. The imaging was performed at the GEMS (Geoanalytical Electron Microscopy and Spectroscopy) facility at the University of Glasgow using a Zeiss Sigma Field Emission (FE) SEM at high vacuum conditions, using accelerating

voltage (EHT) of 5.00 kV, an aperture size of 30 μm, high current mode, and 5.1 mm working distance.

Profilometry. Printed xerogels were prepared as described above. The film thickness was estimated using an Alpha-Step® D-500 Stylus Profiler (KLA Instruments). Measurements were taken 0.5, 1.0, and 1.5 cm along the length of the printed xerogel lines, along the direction of printing.

Nanoindentation. Xerogels were prepared as described above. Nanoindentation measurements were carried out on a Step 700 Surface Testing Platform (UNHT^3, Anton Paar) using a Berkovich indenter tip. Linear loading was applied, with a maximum load of 1000 μN, a loading and unloading rate of 2000 μN/min, and a pause of 60 seconds. The constant force was set at 150 μN, with an approach distance of 7500 nm and an approach and retract speed of 5000 and 2 nm/min, respectively. Prior to each measurement, an adjust depth offset measurement was taken to correctly set up the depth sensor measurement range to eliminate any issues due to film surface inhomogeneities. Measurements were taken 0.5, 1.0, and 1.5 cm along the length of the xerogel, along the direction of printing. Each measurement was performed in triplicate, using a simple matrix where three points in the film were measured with a gap of 1 mm from each other to prevent any surface deformation from the previous indent affecting the next measurement. For each new triplicate, a different area of the film was chosen to again avoid influence from the previous indentation.

FTO windows. FTO-coated glass (50 x 50 x 2.2 mm, TEC 7, surface resistivity ~7 Ω/sq, Sigma Aldrich) was cleaned by submerging in detergent and deionised water, acetone, and isopropanol, respectively, with ultrasonication for 15 minutes in each solvent. Gloves were used throughout to prevent fingerprints on the glass. Gels were prepared as described above but using a 0.1 M NaCl background electrolyte (10% 0.1 M NaCl, aqueous). Gels were formed or printed in an O-ring with a diameter of 30 mm between two pieces of FTO-coated glass facing inwards and held in place with bulldog clips. Copper tape was placed on opposite ends of the top and bottom pieces of glass as working and counter electrodes.

Cyclic voltammetry. Cyclic voltammetry measurements were performed with a Palmsens4 potentiostat. Samples were prepared in the FTO-coated glass assembly as described above. All data were collected using a two-electrode set-up. Therefore, values cannot be stated in reference to a known value. Thus, CVs were carried out using a solution of 10 mg/mL hydroquinone dissolved in water with 10% 0.1 M NaCl in the same set-up. Such data is tabulated in Table S1. For **Gel-1** and **Printed Gel-1**, the potential was scanned from +1.0 V to -1.5 V at a scan rate of 0.05 V/s, and measurements were carried out in triplicate. The clearest of the three scans was used for analysis.

Rheology. Dynamic rheological measurements were performed with an Anton Paar Physica MCR301 rheometer. A cup-and-vane measuring system was used for strain and frequency sweeps.

Gels were prepared as described above. The temperature was maintained at 25°C during all measurements using a water bath. All measurements were recorded in triplicate.

Strain sweeps were formed over a range of 0.1-1000% with a frequency of 10 rad/s. The critical strain (yield point) was quoted where G′ departed from linearity and ultimately crossed over G″ (flow point), causing the gel to break down.

Frequency sweeps were performed from 1 rad/s to 100 rad/s under a strain of 0.1%. G′ and G″ were quoted at 10 rad/s. The measurements were performed within the viscoelastic region where G′ and G″ were independent of strain amplitude.

Flexibility tests. Xerogels were prepared by drop casting or printing onto A4 Acetate Universal Inkjet Transparency Film (Haber Craft, t_s = 120 μm), which was cut into uniform 5.5 x 2.5 cm rectangles, and drying in air. Two silver electrodes were made from silver paste (Agar Scientific, Acheson Silver Electrodag 1415), ensuring proper contact was made to opposite sides of the sample square. Two pieces of copper tape (RS Components) were then attached to the silver electrodes to allow connection to the potentiostat. The xerogels were tested by clamping them on a custom-made motorised moving stage and using a two-point bending setup.[5] Chronoamperometric measurements were simultaneously performed using a Palmsens4 potentiostat and a custom-made data acquisition unit, which collected both the current produced and the displacement of the stage from the starting point. Before each test, the films were irradiated for 5 minutes with a 365 nm LED light (LZ1-10UV00, LED Engin), placed 1 cm away from the sample. The distance from the LED to the sample was adjusted

so that an optical power probe (Thorlabs PM100D with S120VC sensor) placed at the same distance as the sample registered 20 mW of incident power. The stage was moved 4 cm away from the starting point in increments of 0.5 cm at a constant linear loading speed of 5 mm/s. The film was held at each position for 1 minute, and the current measured. The stage was then moved back to the initial starting point, again in 0.5 cm increments for 1 minute, and again the current was measured at each position. Tracing the film outline in digital side view photographs of the film allowed ImageJ software to calculate the bending angle.

For bending cycles, samples were subjected to continuous cyclic loading (no dwell time between cycles) with a linear displacement of 2.5 cm. Each full cycle, comprising the bending and relaxation phases, was completed in 10 seconds (corresponding to a frequency of 0.1 Hz). This process was repeated for 1,000 consecutive cycles.

Bending strain. To precisely map the mechanical severity of the deformation, the bending strain (ε) accommodated by the active layer was derived using the expression $\varepsilon = \pm h/r$. Here, h represents the distance from the structural neutral axis to the centreline of the printed functional films. Assuming negligible mechanical contribution from the **PBI-A/PEO** film, the neutral axis sits exactly in the geometric centre of the acetate film. The thickness of the acetate film (t_s) and **PBI-A/PEO** film (t_f) are 120 and 16.6 μm, respectively. Therefore,

Neutral axis location: $y_n = t_s/2 = 120\ \mu m/2 = 60\ \mu m$.

Centre of printed film: $y_f = t_s + (t_f/2) = 120\ \mu m + (16.6\ \mu m/2) = 128.3\ \mu m$.

$h = y_f - y_n = 128.3\ \mu m - 60\ \mu m = 68.3\ \mu m$.

Following established protocols, the radius of curvature (r) at each discrete deformation position was evaluated with its relationship with the chord length (c), defined as $c = 2r \sin(l/2r)$, where l is the arc length of the bilayer (55 mm).[6]

Linear sweep voltammetry. Linear sweep measurements were recorded from -4 to +4 V at a scan rate of 0.05 V s^{-1} using a Plamsens4 Potentiostat with a two-electrode setup. Films were measured before and after bending cycles. Bending cycles were completed as described above. The counter and reference electrodes were connected to one piece of copper tape, and the working electrode was attached to the other. The samples were irradiated with a 365 nm LED light (RS Components Ltd) and placed 1 cm away from the sample during measurements. The distance from the LED to the sample was adjusted so that an optical power probe (Thorlabs PM100D with S120VC sensor) placed at the same distance as the sample registered 20 mW of incident power. All samples were recorded in triplicate.

Supplementary Figures and Tables

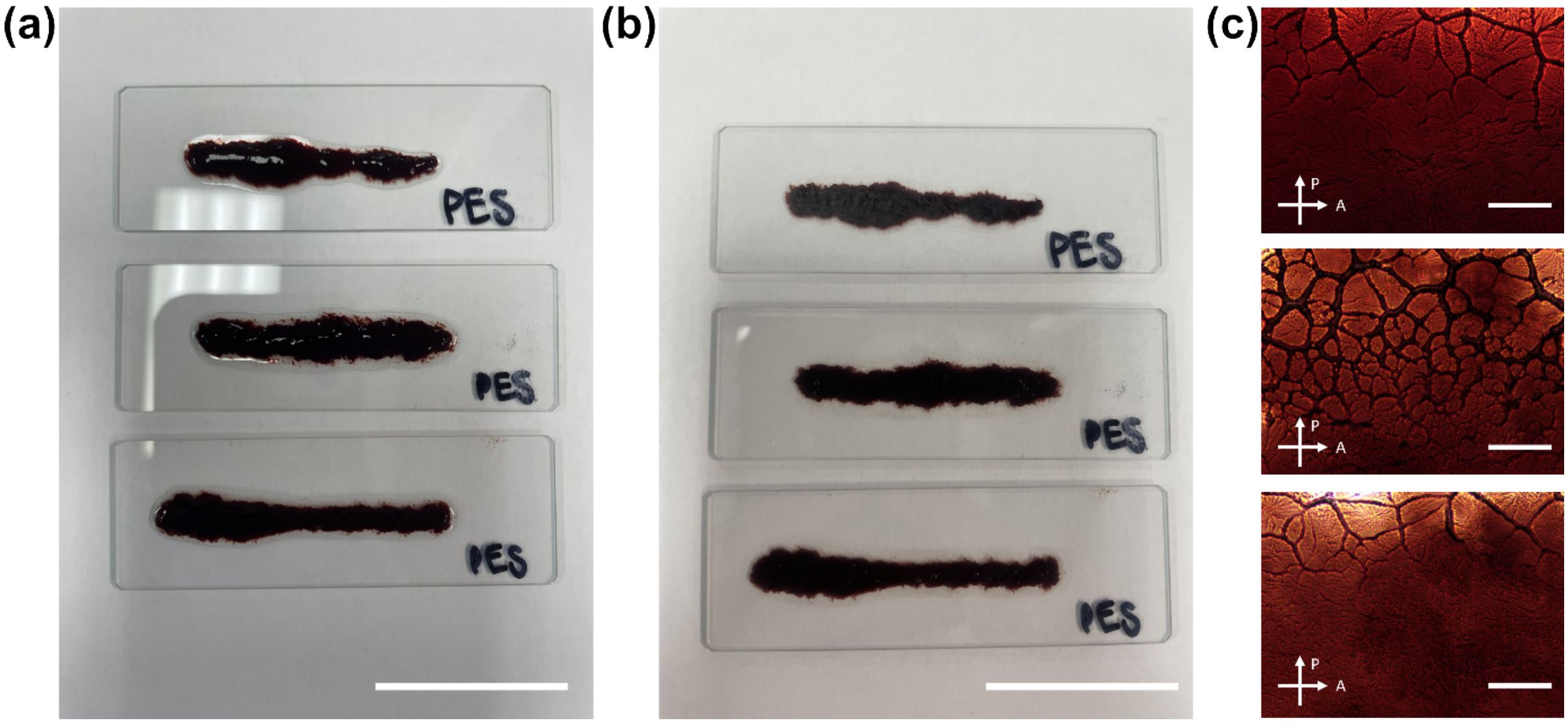

Figure S1. Photographs of (a) printed **PBI-A**/PES blends and (b) their corresponding xerogels. (c) Cross-polarised optical microscope images of printed xerogels. Scale bars represent 100 μm for optical microscope images and 2 cm for photographs.

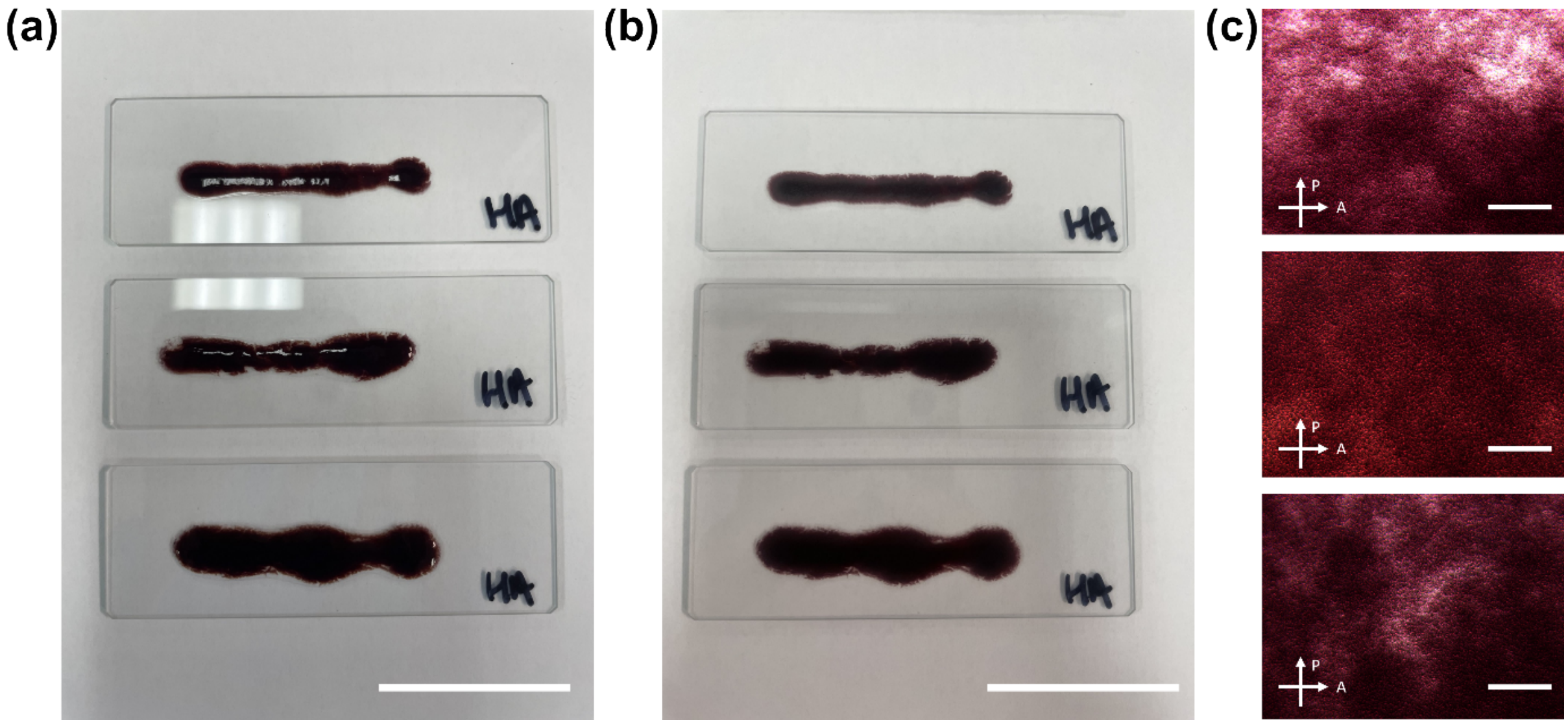

Figure S2. Photographs of (a) printed PBI-A/HA blends and (b) their corresponding xerogels. (c) Cross-polarised optical microscope images of printed xerogels. Scale bars represent 100 μm for optical microscope images and 2 cm for photographs.

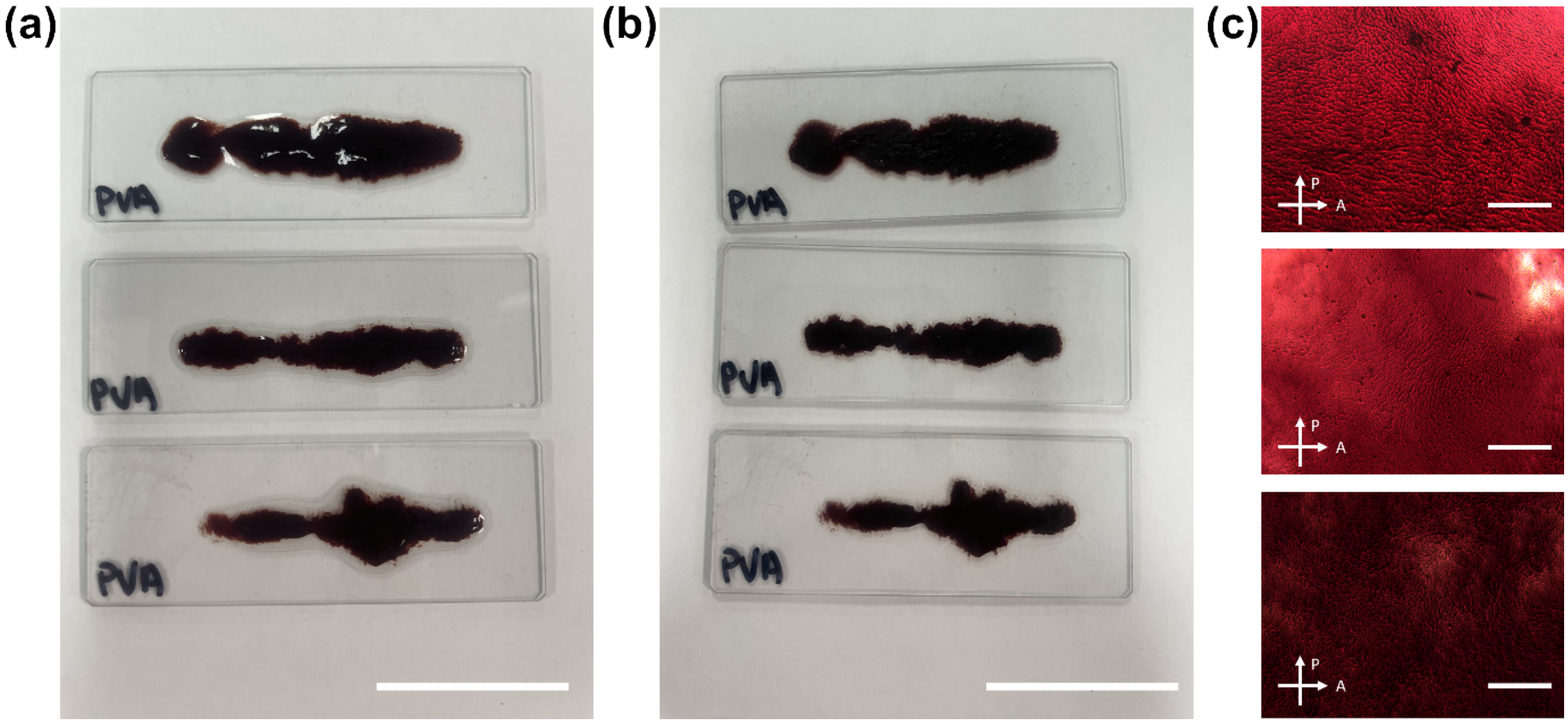

Figure S3. Photographs of (a) printed PBI-A/PVA blends and (b) their corresponding xerogels. (c) Cross-polarised optical microscope images of printed xerogels. Scale bars represent 100 μm for optical microscope images and 2 cm for photographs.

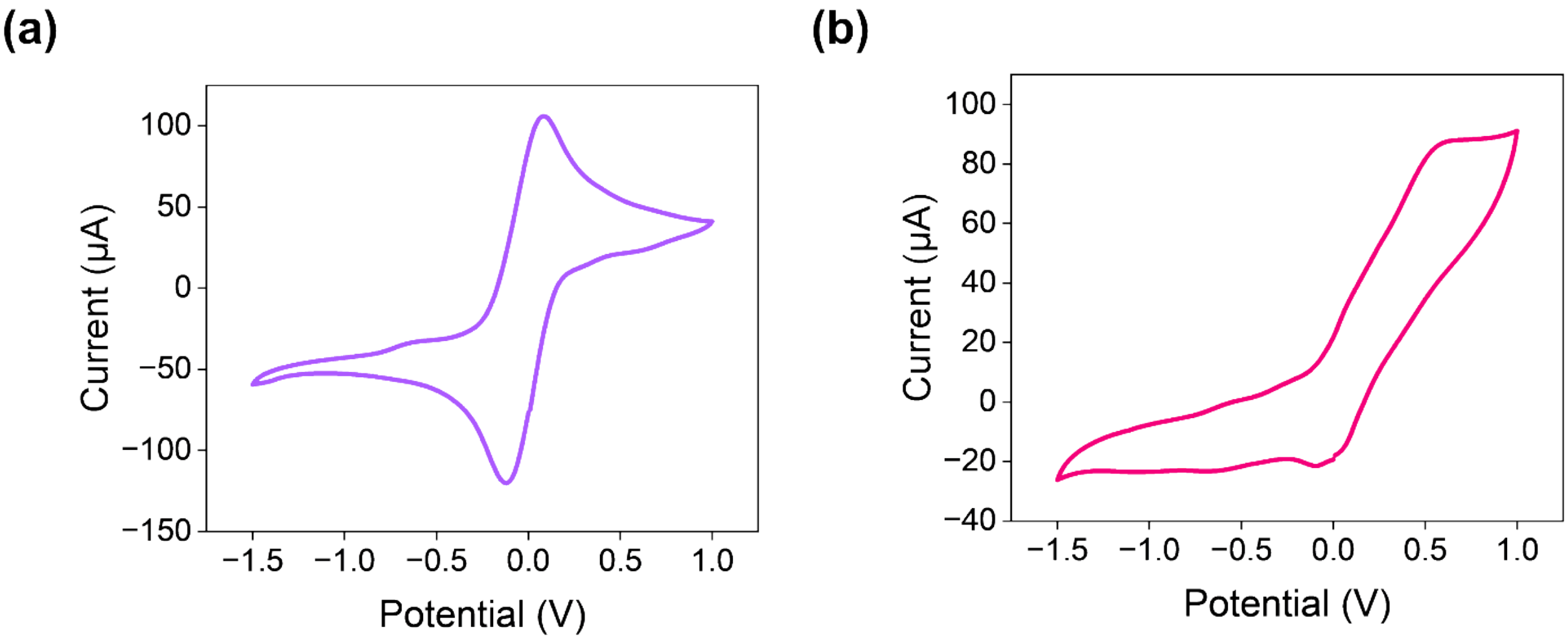

Figure S4. Cyclic voltammograms of (a) **Gel-1** and (b) **Printed Gel-1** using 0.1 M NaCl (aq) electrolyte solution with a scan rate of 0.05 V/s.

Table S1. Reduction and oxidation potentials taken from cyclic voltammograms of 10 mg/mL hydroquinone with 10% 0.1 M NaCl. Cyclic voltammograms were performed in a 1 x 1 cm FTO window set-up. The scan rate of the cyclic voltammograms were 0.5 V/s.

Control	Reduction potentials (V)	Oxidation potential (V)
1 x 1 cm FTO window	-1.3, -2.0	0.7

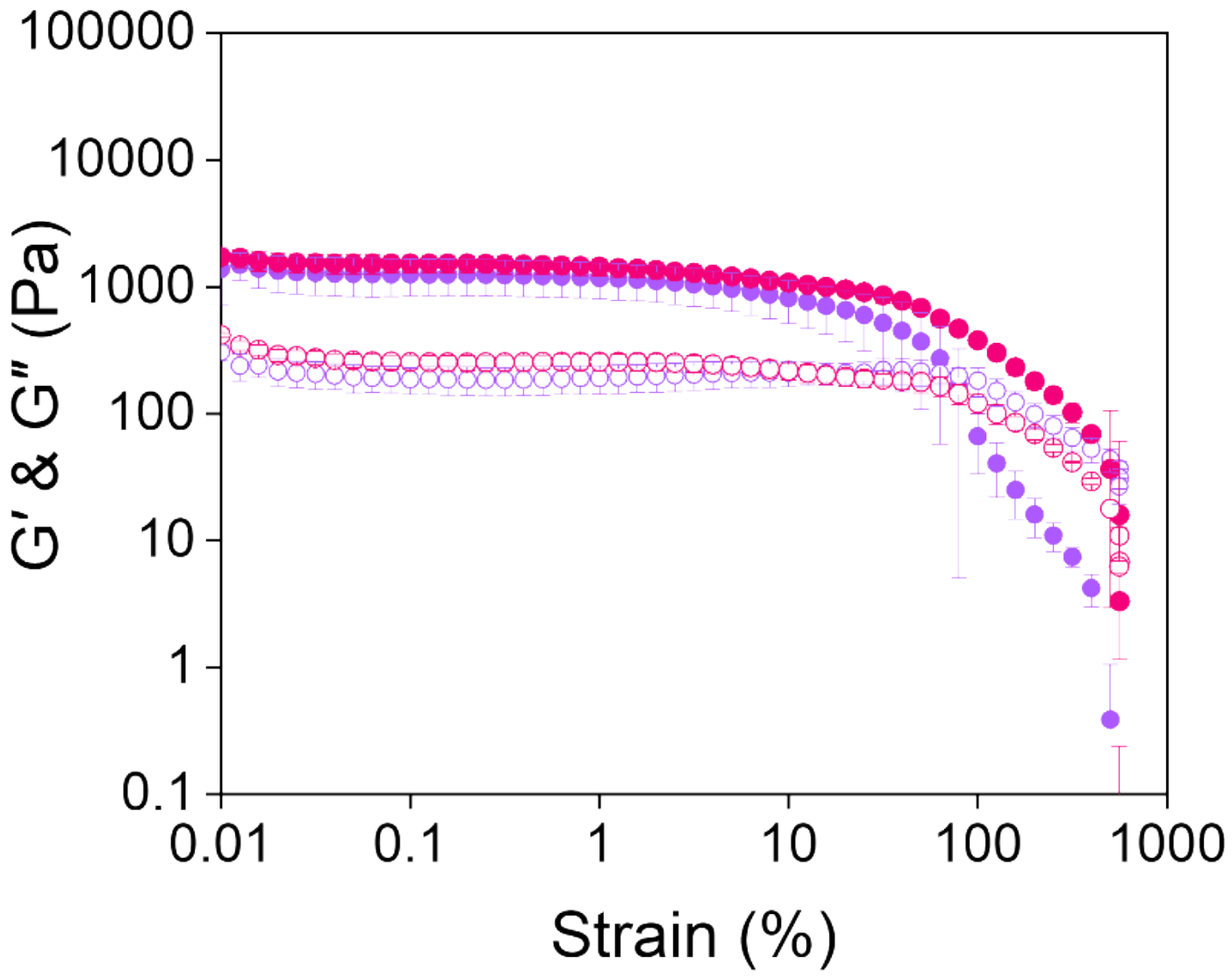

Figure S5. Strain sweep of **Gel-1** without (purple) and with (pink) 1 equivalent of NaCl (0.1 M, aqueous). Closed circles represent G′ and open circles represent G″. Strain sweeps were performed at a frequency of 10 rad/s at 25°C. Data shown are averaged data for triplicate runs of the samples, with error bars representing standard deviation.

Figure S6. Frequency sweep of **Gel-1** without (purple) and with (pink) 1 equivalent of NaCl (0.1 M, aqueous). Closed circles represent G′ and open circles represent G″. Frequency sweeps were performed at a strain of 0.1% at 25°C. Data shown are averaged data for triplicate runs of the samples, with error bars representing standard deviation.

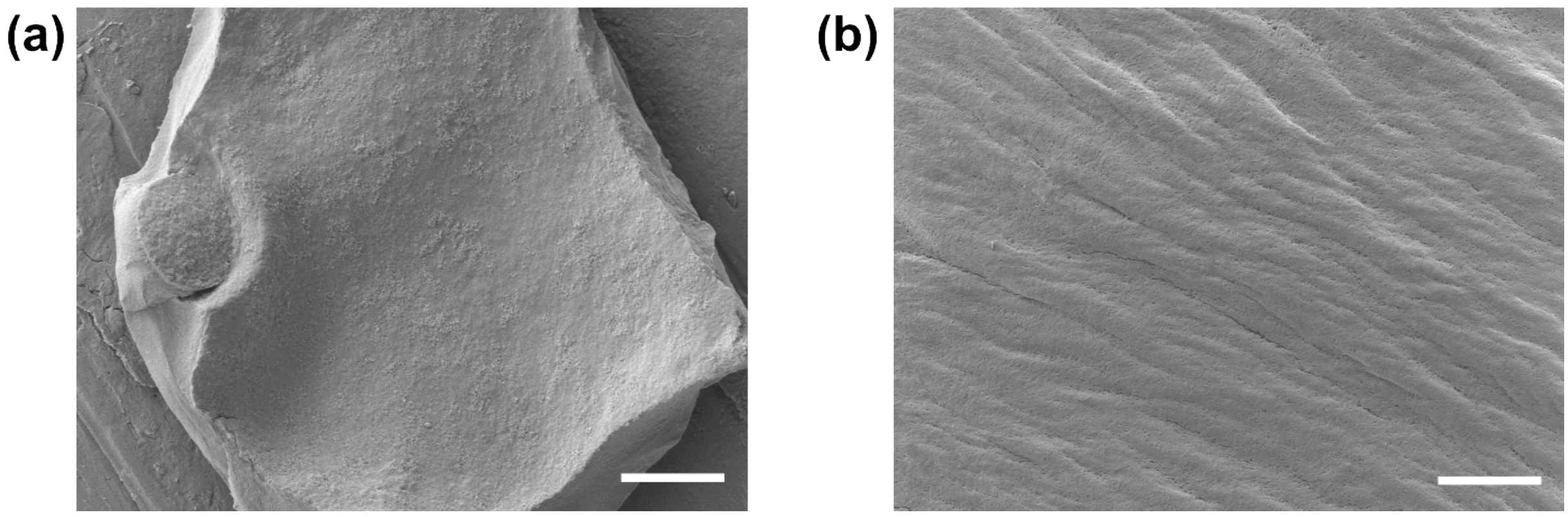

Figure S7. SEM images of (a) **Gel-1** and (b) **Printed Gel-1** at 2.00 kx magnification. Scale bar represents 10 μm.

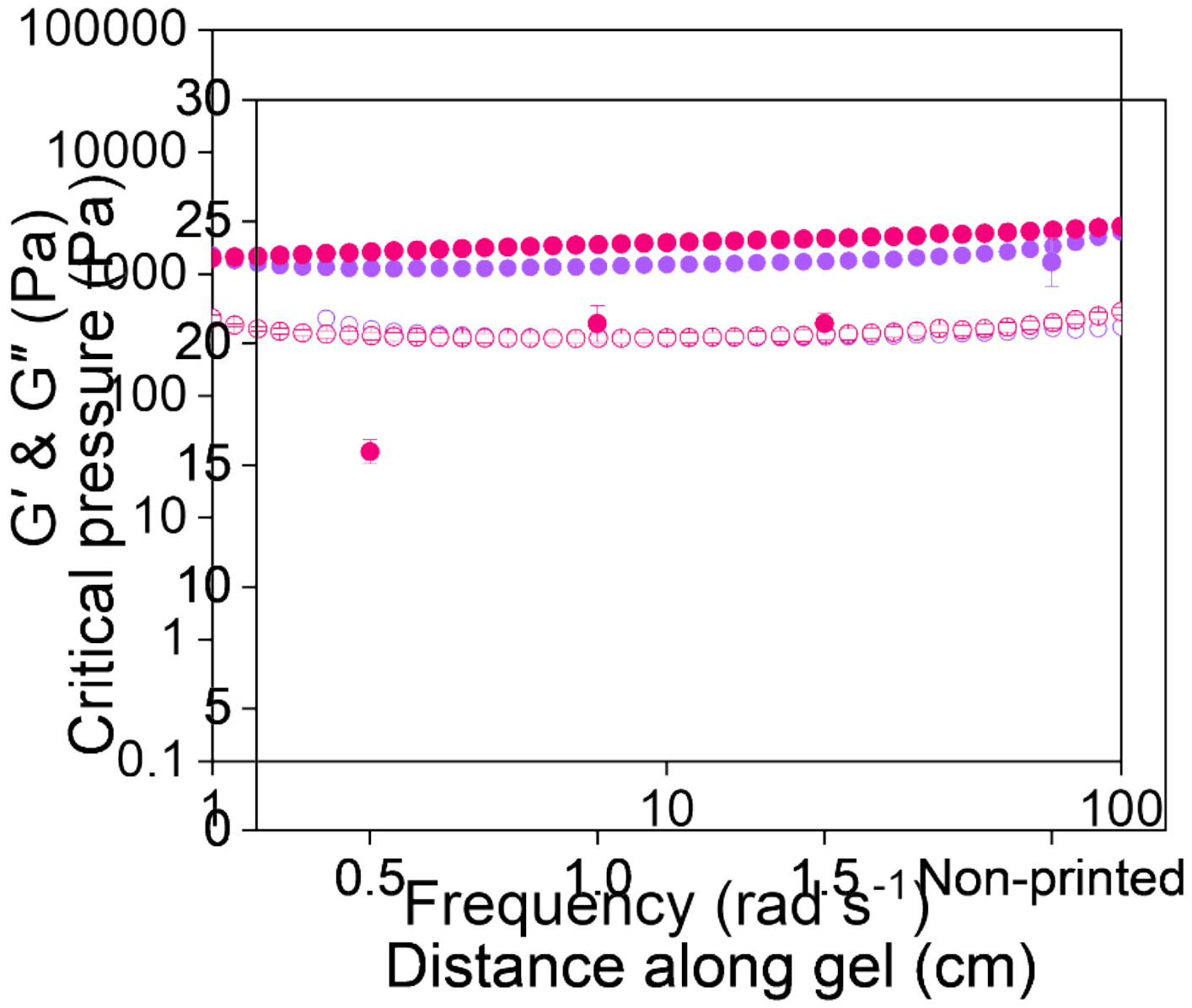

Figure S8. Cavitation rheology of **Gel-1** (purple) and **Printed Gel-1** (pink). Data shown are averaged data for triplicate runs, with error bars representing standard deviation.

Table S2. Tabulated thickness of the **Printed Gel-1** xerogel along the printing axis.

Distance along gel (cm)	Thickness (μm)
0.5	16.76
1.0	16.83
1.5	16.61

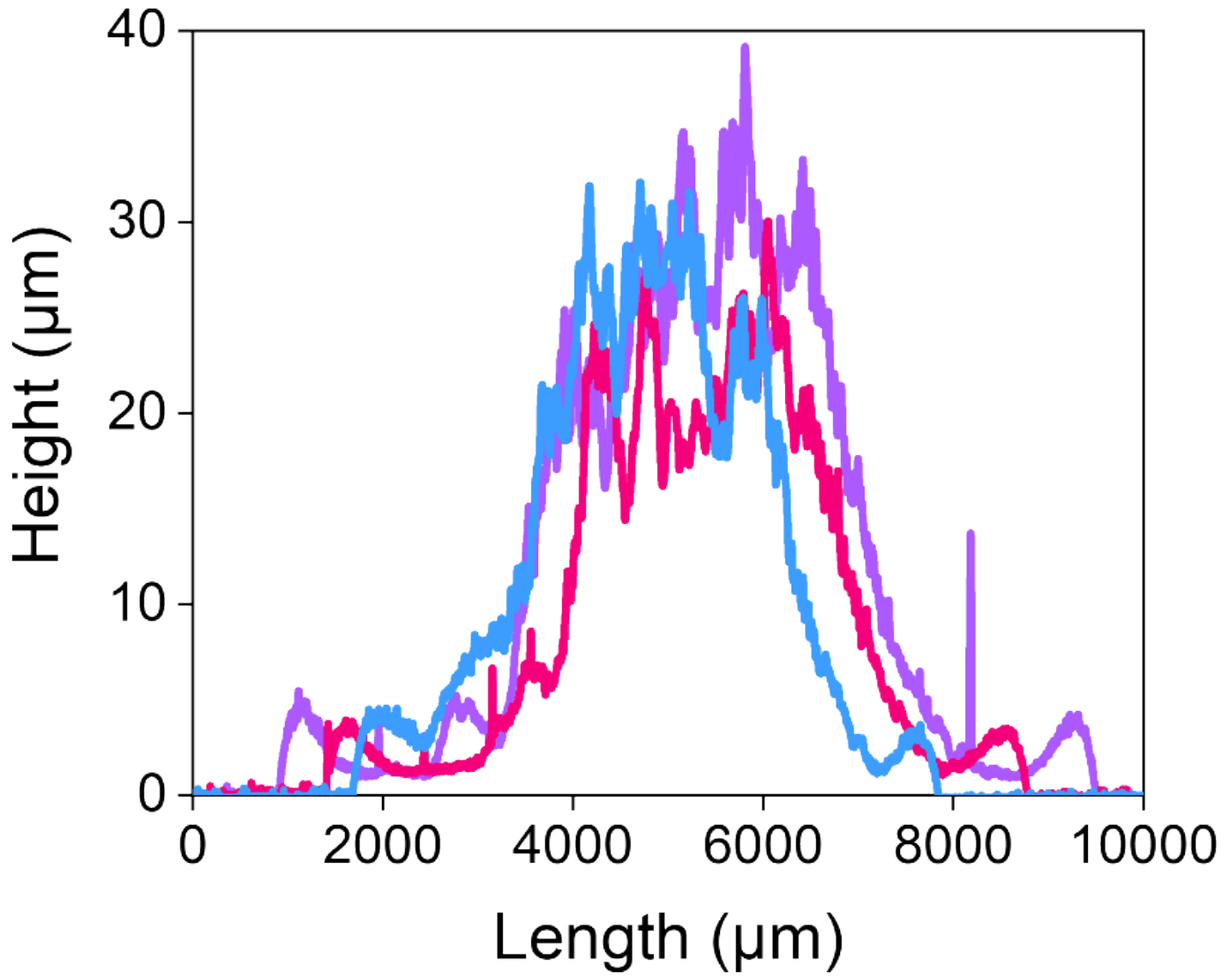

Figure S9. Profilometry profile of **Printed Gel-1** xerogels measured 0.5 cm (purple), 1.0 cm (pink), and 1.5 cm (blue) along the printing axis.

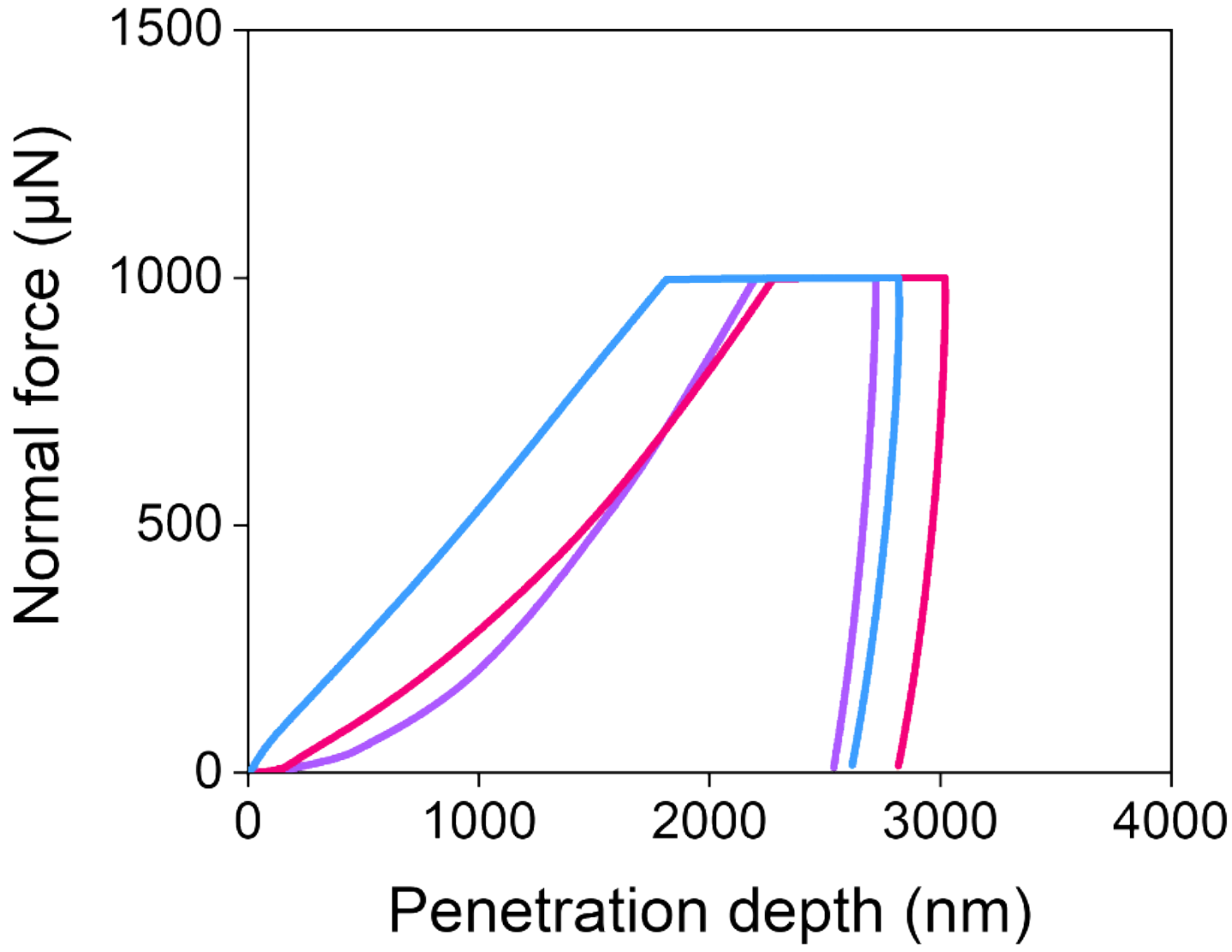

Figure S10. Force-penetration depth curve obtained during nanoindentation of **Printed Gel-1** xerogels measured 0.5 cm (purple), 1.0 cm (pink), and 1.5 cm (blue) along the printing axis.

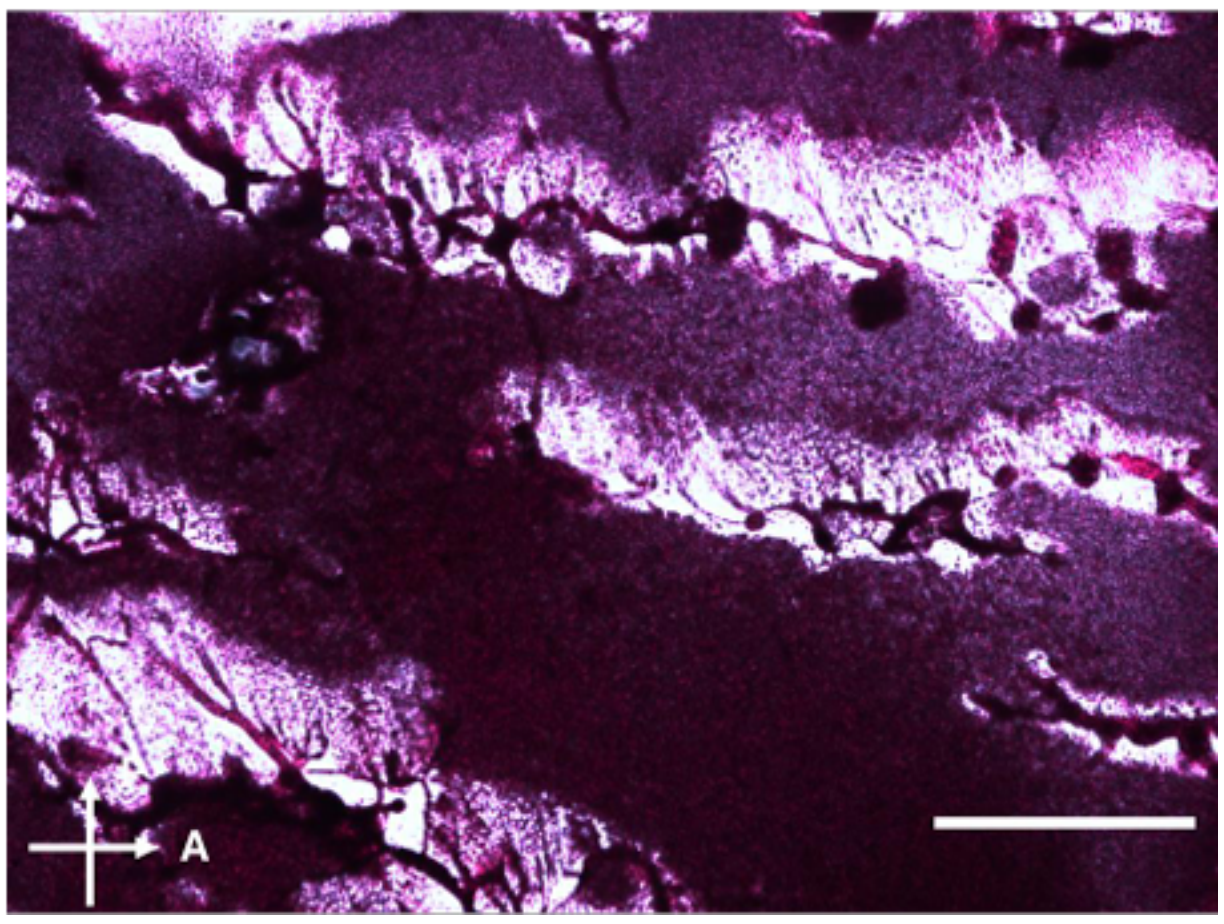

Figure S11. Cross-polarised optical microscope images of a **Gel-1** xerogel. Scale bar represents 100 μm.

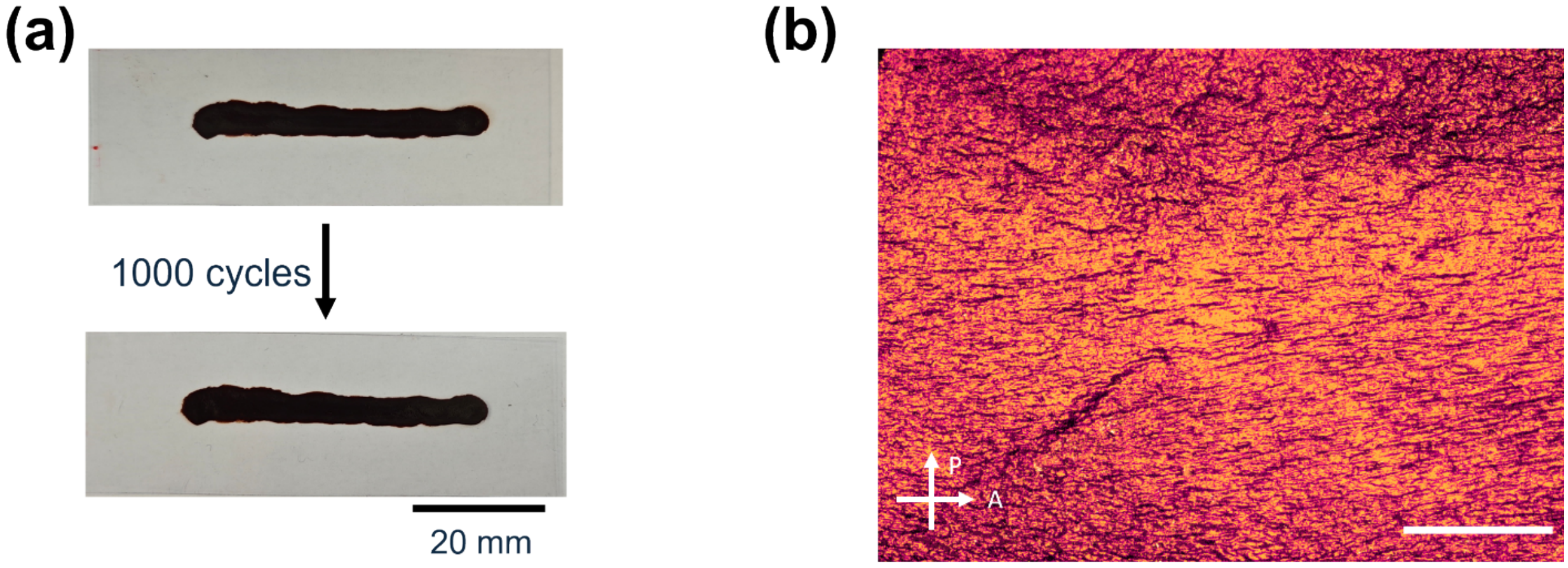

Figure S12. (a) Photographs of **Printed Gel-1** xerogel before (top) and after (bottom) 1000 bending cycle. Scale bar represents 20 mm. (b) Cross-polarised optical microscope images of Printed Gel-1 xerogel after 1000 bending cycle. Scale bar represents 100 μm.

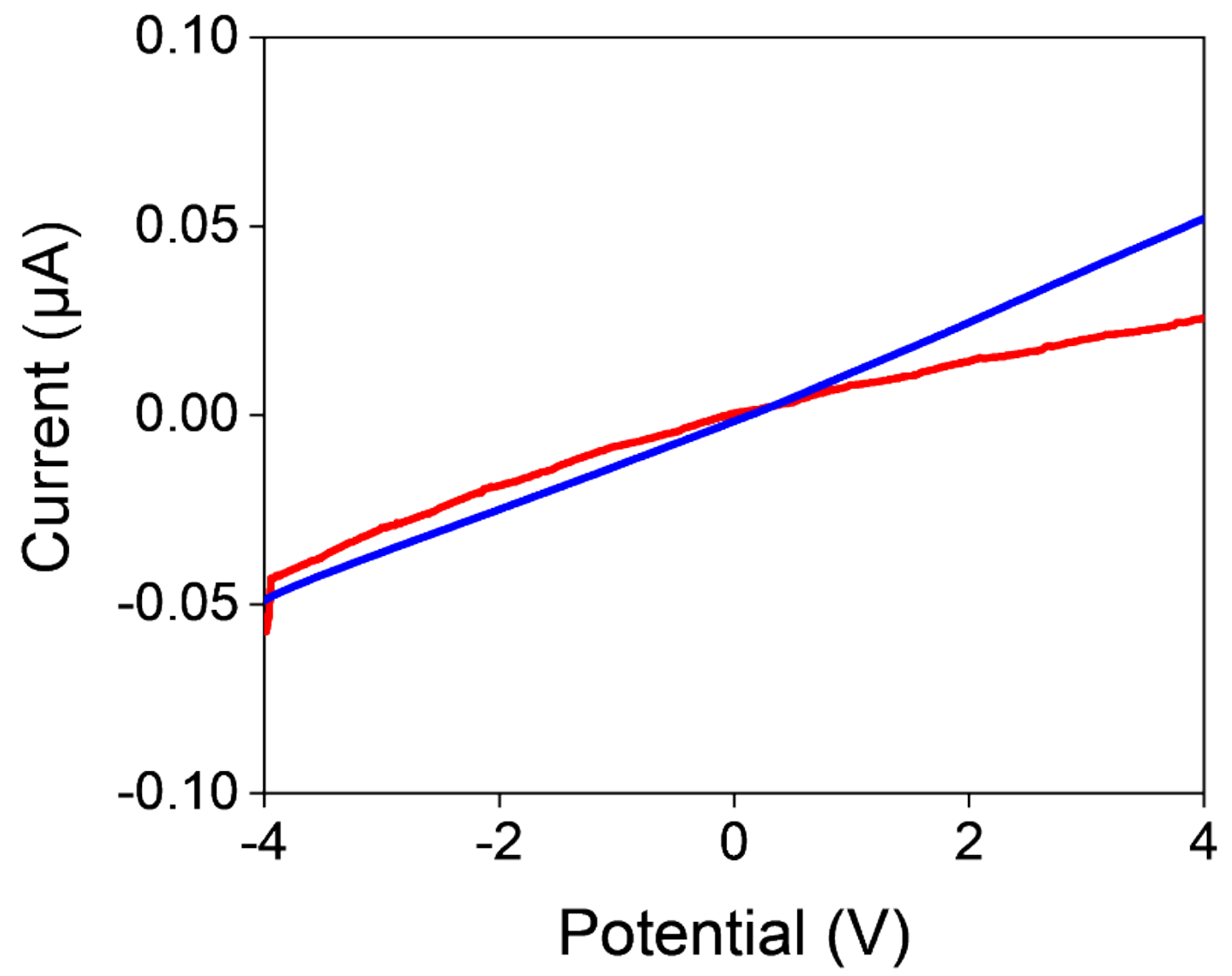

Figure S13. IV plots showing the photoresponse of **Printed Gel-1** xerogels before (blue) and after (red) 1000 bending cycles. Data shown are averaged for triplicate samples.

References

1 E. R. Draper, J. J. Walsh, T. O. McDonald, M. A. Zwijnenburg, P. J. Cameron, A. J. Cowan and D. J. Adams, *J. Mater. Chem. C*, 2014, **2**, 5570–5575.
2 M. C. Nolan, A. M. Fuentes Caparrós, B. Dietrich, M. Barrow, E. R. Cross, M. Bleuel, S. M. King and D. J. Adams, *Soft Matter*, 2017, **13**, 8426–8432.
3 A. M. Fuentes-Caparrós, B. Dietrich, L. Thomson, C. Chauveau and D. J. Adams, *Soft Matter*, 2019, **15**, 6340–6347.
4 L. Thomson, R. Schweins, E. R. Draper and D. J. Adams, *Macromol. Rapid Commun.*, 2020, **41**, 2000093.
5 M. Nankali, N. M. Nouri, M. Navidbakhsh, N. Geran Malek, M. A. Amindehghan, A. Montazeri Shahtoori, M. Karimi and M. Amjadi, *J. Mater. Chem. C Mater.*, 2020, **8**, 6185–6195.
6 M. Amjadi and M. Sitti, *Adv. Sci.*, 2018, **5**, 1800239.
